# The application of 3D-printed auto-stable artificial vertebral body in en bloc resection and reconstruction of thoracolumbar metastases

**DOI:** 10.1186/s13018-023-04135-3

**Published:** 2023-08-29

**Authors:** Yun Cao, Nan Yang, Shengbao Wang, Cong Wang, Qiang He, Qinfan Wu, Yangyang Zheng

**Affiliations:** Department of Spine, Sichuan Science City Hospital, Mianyang, Sichuan Province China

**Keywords:** 3D-printed auto-stable artificial vertebra, Thoracolumbar metastases, En bloc resection

## Abstract

**Background:**

Nerve compression symptoms and spinal instability, resulting from spinal metastases, significantly impact the quality of life for patients. A 3D-printed vertebral body is considered an effective approach to reconstruct bone defects following en bloc resection of spinal tumors. The advantage of this method lies in its customized shape and innermost porous structure, which promotes bone ingrowth and leads to reduced postoperative complications.

**Objective:**

The purpose of this study is to assess the effectiveness of 3D-printed auto-stable artificial vertebrae in the en bloc resection and reconstruction of thoracolumbar metastases.

**Methods:**

This study included patients who underwent en bloc resection of thoracolumbar metastases based on the Weinstein-Boriani-Biagini surgical staging system, between January 2019 and April 2021. The patients were divided into two groups: the observation group, which was reconstructed using 3D-printed auto-stable vertebral bodies, and the control group, treated with titanium cages and allograft bone. Evaluation criteria for the patients included assessment of implant subsidence, instrumentation-related complications, VAS score, and Frankel grading of spinal cord injury.

**Results:**

The median follow-up period was 21.8 months (range 12–38 months). Among the patients, 10 received a customized 3D-printed artificial vertebral body, while the remaining 10 received a titanium cage. The observation group showed significantly lower operation time, intraoperative blood loss, and postoperative drainage compared to the control group (*P* < 0.05). At the final follow-up, the average implant subsidence was 1.8 ± 2.1 mm for the observation group and 5.2 ± 5.1 mm for the control group (*P* < 0.05). The visual analog scale (VAS) scores were not statistically different between the two groups at preoperative, 24 h, 3 months, and 1 year after the operation (*P* < 0.05). There were no statistically significant differences in the improvements of spinal cord functions between the two groups.

**Conclusion:**

The utilization of a 3D-printed auto-stable artificial vertebra for reconstruction following en bloc resection of thoracolumbar metastases appears to be a viable and dependable choice. The low occurrence of prosthesis subsidence with 3D-printed prostheses can offer immediate and robust stability.

## Introduction

Spinal tumors encompass primary tumors originating in the spine and metastatic tumors arising from other sites [1]. The spine is the most common site of bone metastasis for malignant tumors, with significantly higher incidence compared to primary tumors. Spinal metastases occur in about 30–70% of patients with malignant tumors [2, 3]. These metastases can lead to severe back pain, limb numbness, weakness, and/or paralysis. Spinal cord compression affects 5–10% of patients with spinal metastases [4, 5], greatly impacting patients' quality of life, survival, and placing a substantial burden on society.

Treatment options for spinal metastases include radiotherapy, chemotherapy, surgery, and biological therapy. Surgery is a crucial approach for spinal tumors [6]. En bloc resection is a widely used surgical method, aiming to completely remove tumors with sufficient margins. When the stability of the spine is disrupted and the spinal cord nerves are compressed by tumors, en bloc resection is feasible. The choice of intraoperative implants plays a pivotal role in the surgery. Conventional implants for reconstruction include titanium cages, artificial vertebrae, autologous bone, and bone cement combined with an anterior nail plate or posterior pedicle screw system. However, these implants have drawbacks, such as lack of individualization, long operation time, and increased bleeding. Additionally, issues like internal fixation loosening and breakage are not uncommon, leading to postoperative spinal instability. 3D printing technology, a product of advancements in computer technology and image digitization, offers unique advantages in orthopedic implant design and manufacturing. In spinal surgery, the 3D-printed auto-stable vertebra closely matches the upper and lower vertebral bodies, addressing anatomical and physiological demands while providing immediate postoperative spinal stability with fewer instrumentation-related complications.

Therefore, this study retrospectively analyzes clinical data from patients with thoracolumbar metastases treated using either 3D-printed auto-stable artificial vertebrae or titanium cages. The aim is to evaluate the efficacy of 3D printing technology in treating spinal metastases.

## Materials and methods

### Study design and population

This study involved a retrospective analysis conducted at a single center. It received approval from the institutional review board of the ethics committee, and all patients provided written informed consent. The study included a total of 20 patients with thoracolumbar metastases who underwent en bloc resection between January 2019 and April 2021. These patients were divided into two groups: the observation group (using 3D-printed auto-stable artificial vertebral bodies) and the control group (not using 3D-printed implants). Each group comprised 10 patients. The inclusion criteria were as follows: (1) Confirmed solitary thoracolumbar metastases through histological diagnosis; (2) Postoperative follow-up exceeding 12 months with available imaging data; (3) Tomita score less than 4 and Tokuhashi score more than 8; (4) Presence of nerve compression symptoms or aggressive bone damage. Patients with extensive visceral metastases or those unable to tolerate general anesthesia and surgery were excluded from the study. The patient characteristics and treatment-related data were gathered from electronic medical records. Imaging data, including radiographs, CT scans, MRI images, and emission computed tomography, were obtained from the picture archiving and communication system. The patients who underwent surgery were regularly followed up.

### 3D-printed auto-stable artificial vertebrae

Similar to the approach used in our previous study [7], the observation group utilized Mimics 17.0 software to process the preoperative imaging examination's DICOM format files for prosthesis design. A 3D model was established through computer calculations, and 3D reconstruction images were obtained via image fusion technology. The target areas encompassed tumors, bones, blood vessels, and nerves. The models were created using multi-segmentation masks of different colors, restoring bones with corresponding blood vessels and nerves to register the 3D model. A 1:1 multisegment solid spinal tumor model was 3D-printed using polylactic acid. Additionally, the 3D-printed auto-stable artificial vertebrae featured a porous surface and innermost scaffold structure, mimicking cancellous bone, which allowed for bony ingrowth and regulation of elastic modulus. The prosthesis had pore diameters of 700 ± 80 µm and wire diameters of 300 ± 100 µm, with an average porosity of 73%.

**Fig. 1:**
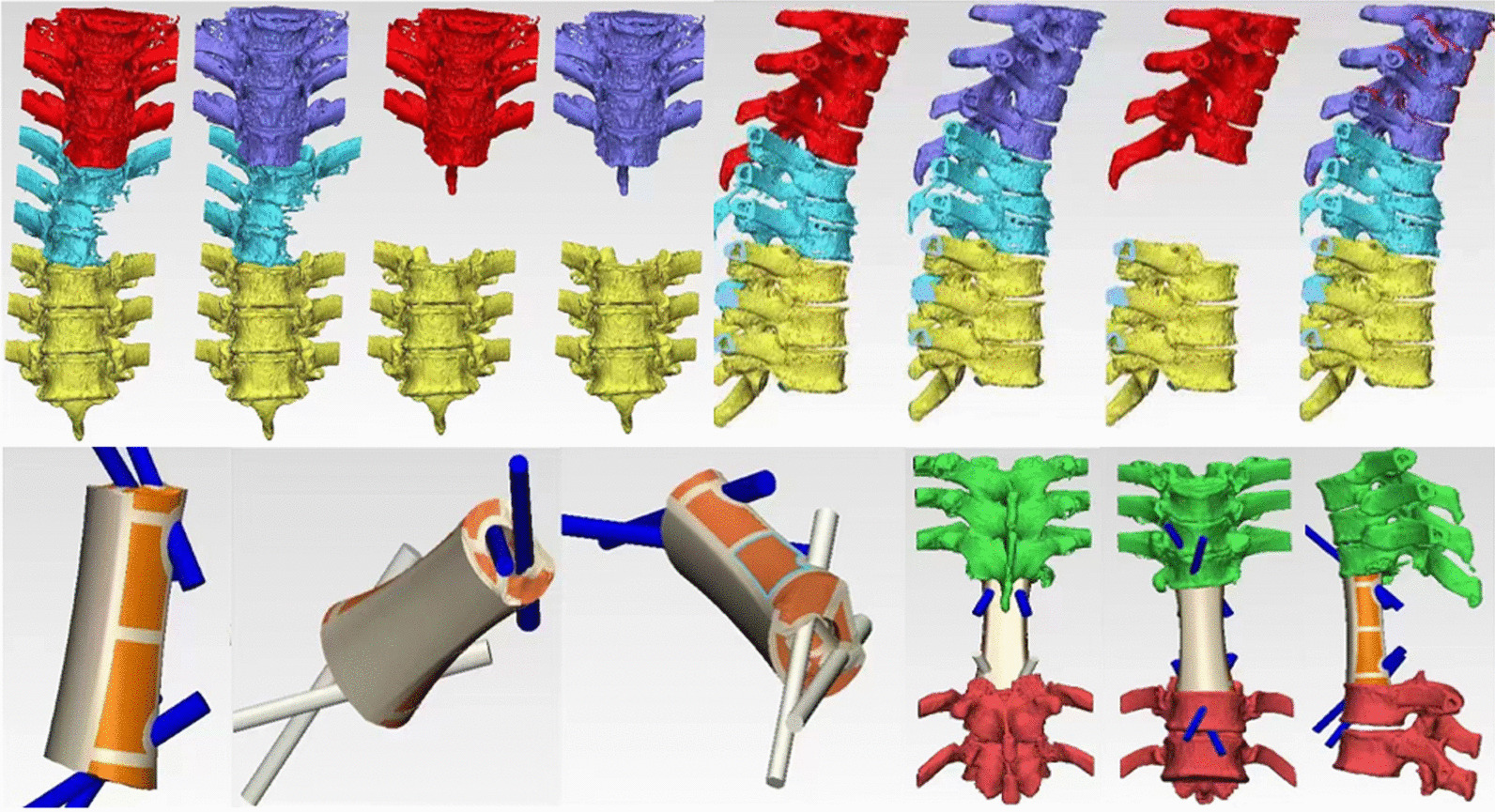
3D-printed auto-stable artificial vertebrae

To determine the best insertion point and corresponding needle catheter, the spinal vertebral model and corresponding nail channel data were imported into 3-matic 9.0 reverse engineering software. A reverse template was constructed in conjunction with the injection catheter to form a personalized needle guide plate. Subsequently, the pin guide plate underwent in vitro verification and was handled through low-temperature plasma disinfection. Due to the varying organs located in front of different spinal segments, personalized perioperative treatment measures and surgical plans were developed. The 3D printing model accurately depicted the surgical area's anatomical structure, allowing for precise determination of the lesion's location and clarification of its morphology and resection scope. Moreover, the 3D model of the guide plate facilitated accurate screw insertion, effectively simulating the angle and depth of the nail inlet, providing guidance for surgical screw selection (Fig. 1).

### Surgical procedure

For preoperative evaluation, X-ray, CT, MRI, and positron emission CT (PET-CT) scans were routinely conducted. Additionally, when tumors closely involved major vascular structures, CT angiography was performed. In both groups, all patients received intravenous general anesthesia with endotracheal intubation, followed by tumor resection via a posterior approach and internal fixation. During the procedure, the surgeon implanted the 3D-printed prosthesis in the observation group and performed posterior reconstruction using pedicle screws and transverse connectors. The posterior instrumentation was slightly adjusted to compress the inserted prosthesis. On the other hand, in the control group, the surgeon performed the procedure using a titanium cage with autologous iliac bone implantation.

### Observed outcomes

Implant subsidence was assessed on midsagittal reconstructed CT images and quantified as the loss of segment height. This measurement involved determining the distance between the midpoint of the upper endplate of the vertebral body above the resection site and the midpoint of the lower endplate of the vertebral body below the resection site. Prosthesis subsidence was defined as a reduction in segment height from the immediate postoperative measurement to the last follow-up measurement. Fusion time was determined using CT imaging. To provide evidence of osseointegration at the bone-metal contact surfaces, we analyzed the density change inside the prosthesis by measuring CT HU. For CT HU measurement, four 2 × 2 mm areas were selected on the upper and lower ends of the prosthesis and both sides of the prosthesis. The average value of these measurements was taken as the patient's postoperative CT HU value.

We evaluated the VAS score and Frankel grading at preoperative, 24 h, 3 months, and 1 year after the operation. Additionally, we reviewed all postoperative imaging to identify any instrumentation-related complications, such as implant loosening, breakage, prosthesis migration, or other noticeable complications detectable on imaging during the follow-up period.

### Statistical analysis

Statistical calculations were conducted using SPSS version 28.0. Measurements that followed a normal distribution were presented as mean ± standard deviation, and we used the independent sample *t *test for these data. For count data, we employed the chi-square test. A significance level of *P* < 0.05 was considered statistically significant.

## Results

### Demographic and clinical characteristics

Twenty patients underwent en bloc resection of thoracolumbar metastasis. Table 1 provides details of their baseline demographic and clinical characteristics, such as comorbidities, symptoms, duration of symptoms, radiological assessment, and treatments. There were no significant differences in general data between the two groups, including age, height, weight, and histological grade (*P* > 0.05).

**Table 1:** Comparison of general data between the two groups

	Control group (*n* = 10)	Observation group (*n* = 10)	*t*	*P*
Age (yeas)	56.3 ± 16.2	55.4 ± 14.3	1.155	0.099
Sex				> 0.999
Male	4	5		
Female	6	5		
Height (cm)	164.3 ± 10.2	166 ± 11.4.1	0.996	0.078
Weight (kg)	58.1 ± 9.8	59.5 ± 10.2	− 0.684	0.128
Primary tumor				
Mammary cancer	4	3		
Lung cancer	2	2		
Prostate cancer	2	2		
Renal cancer	1	0		
Gastric cancer	1	2		
Colorectal cancer	0	1		
Histological grade	4.3 ± 1.2	4.2 ± 1.7	− 0.933	0.217
Tokuhashi score	8.2 ± 1.5	7.9 ± 1.7	− 0.427	0.675
Frankel classification				
A	0	0		
B	2	2		
C	4	3		
D	4	5		
E	0	0		

The primary site of cancer in the study participants was as follows: breast (7 patients), lung (4 cases), prostate (4 cases), renal (1 case), stomach (3 cases), and colorectum (1 case). Among them, 13 cases had lesions in the thoracic spine, and the remaining 7 cases had lesions in the lumbar spine. Ten patients received 3D-printed artificial vertebral bodies, while the other 10 received titanium cages. The median follow-up for all patients was 21.8 months (range 12–38 months). There was no significant difference in survival between the two groups (*P* > 0.05).

During the perioperative period, the observation group showed significantly lower operation time and intraoperative blood loss compared to the control group (*P* < 0.05). After surgery, the observation group had significantly lower postoperative drainage rates and shorter extubation times than the control group (*P* < 0.05). Refer to Table 2 for detailed data.

**Table 2:** Comparison of surgery in the two groups

	Control group	Observation group	*t*	*P*
Operation time (h)	9.1 ± 3.2	8.1 ± 2.3	1.601	0.021
Intraoperative blood loss (ml)	1850.5 ± 1116.9	1614.3 ± 1052.6	− 0.942	0.044
Postoperative flow was induced (ml)	800.6 ± 206.2	500.8 ± 180.6	1.356	0.033
Extubation time (h)	6.1 ± 3.2	4.3 ± 2.7	− 0.564	0.047

### Comparison of the correlation scores

After surgery, both groups of patients experienced relief from their preoperative symptoms. VAS scores were assessed at preoperative, 24 h, 3 months, and 1 year after the operation. Before surgery, the VAS score for the observation group was 6.3 ± 1.5, and 24 h after surgery, it was 4.9 ± 1.4. There was no statistically significant difference compared to the control group (*t* = – 0.447, *P* = 0.660; *t* = – 0.848, *P* = 0.408). However, at 3 months after surgery, the VAS score for the observation group was 3.3 ± 0.7, and at 1 year after surgery, it was 2.1 ± 1.0 points. The difference was statistically significant compared to the control group (*t* = 0.632, *P* = 0.535; *t* = 0.511, *P* = 0.616) (Table 3).

**Table 3:** Comparison of the correlation scores

	Control group (*n* = 10)	Observation group (*n* = 10)	*t* value	*P* value
VAS				
Preoperative	6.6 ± 1.5	6.3 ± 1.5	− 0.447	0.660
24 h postoperative	5.4 ± 1.3	4.9 ± 1.4	− 0.848	0.408
3 months postoperative	3.1 ± 0.7	3.3 ± 0.7	0.632	0.535
1 year postoperative	1.9 ± 0.7	2.1 ± 1.0	0.511	0.616
Evaluation of spinal function recovery				0.582
Effective	7	9		
Ineffective	3	1		

At the last follow-up, 9 patients in the observation group and 7 patients in the control group regained their spinal function according to the Frankel scale. However, there was no statistical difference in the improvement of spinal cord function between the two groups (*P* = 0.582).

### Comparison of adverse reactions

Both groups were followed after treatment, and the complications were assessed. In the observation group, the occurrences of nerve paralysis, lower limb weakness, hypoesthesia, CSF leak, and infection were all lower compared to the control group (Table 4).

**Table 4:** Comparison of complications after surgery

	Control group	Observation group	*t*	*P*
Weak lower limbs	1	0		> 0.999
Hypaesthesia	3	1		0.582
Leakage of cerebrospinal	7	4		0.370
Infection	1	0		> 0.999

During the postoperative follow-up, the fusion time and implant subsidence in the observation group were lower than those in the control group, and the difference was statistically significant (*P* < 0.05) (Table 5).

**Table 5:** Comparison of the postoperative biofusion conditions

	Control group	Observation group	*t*	*P*
Fusion time	10.9 ± 8.9	12.5 ± 5.2	1.801	0.041
Subsidence of implants	5.2 ± 5.1	1.8 ± 2.1	− 3.011	0.006

## Typical cases

**Fig. 2:**
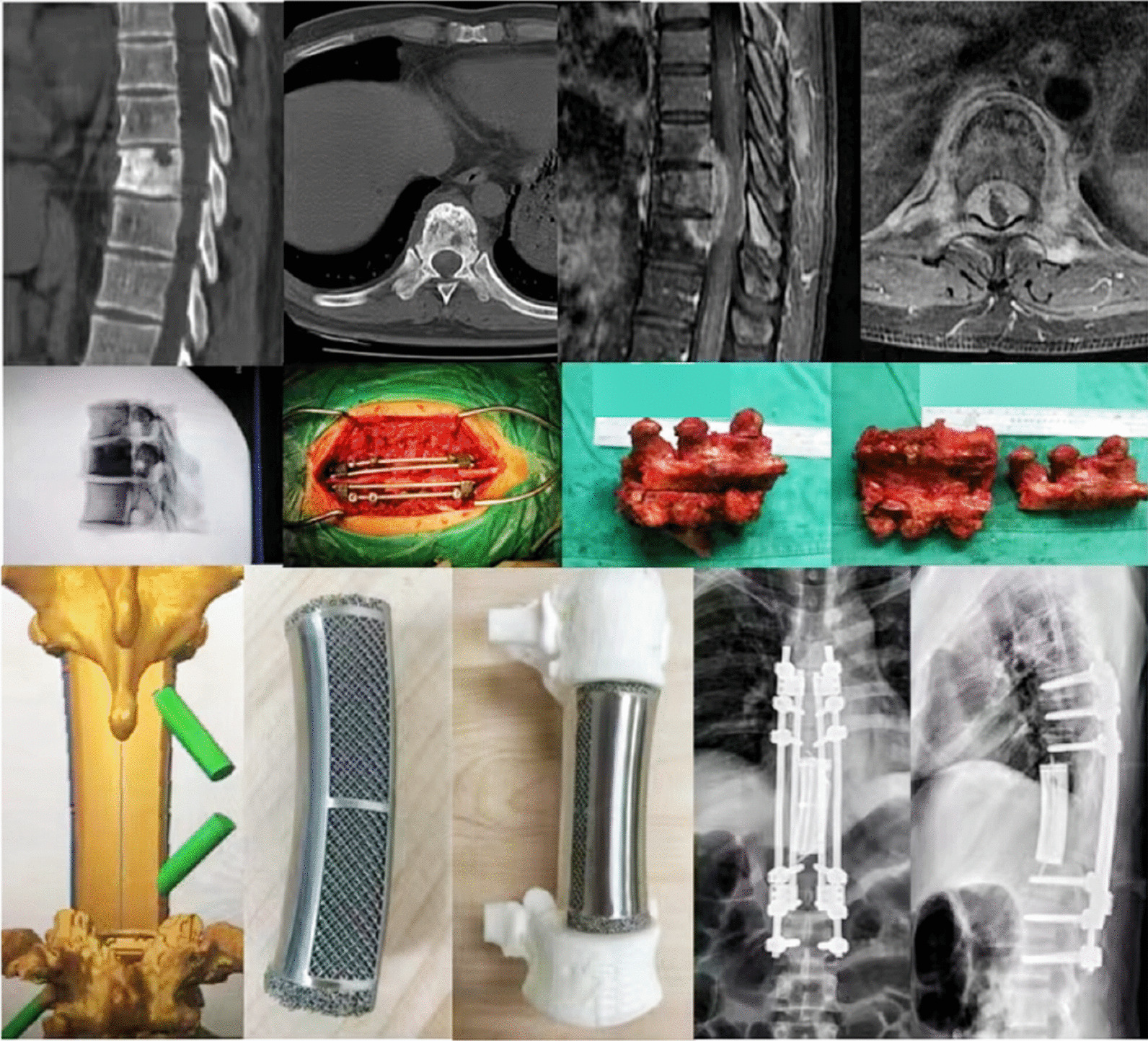
Case 1

*Case* 1: A 36-year-old male was admitted to the hospital with a history of nasopharyngeal cancer, thoracic 10 metastasis, and previous radiotherapy and chemotherapy. He presented with back pain for 3 months, as well as numbness and weakness in both lower extremities for 2 weeks. Examination revealed nerve compression, and the patient's paralysis was progressively worsening. Based on CT, MRI, and other auxiliary examinations, the diagnosis was confirmed as nasopharyngeal cancer spinal metastasis. The patient underwent en bloc resection of the tumor vertebral body with neuroelectrophysiological detection, followed by reconstruction using pedicle screw internal fixation and 3D-printed prosthesis implantation. After surgery, the patient showed a good recovery (Fig. 2).

**Fig. 3:**
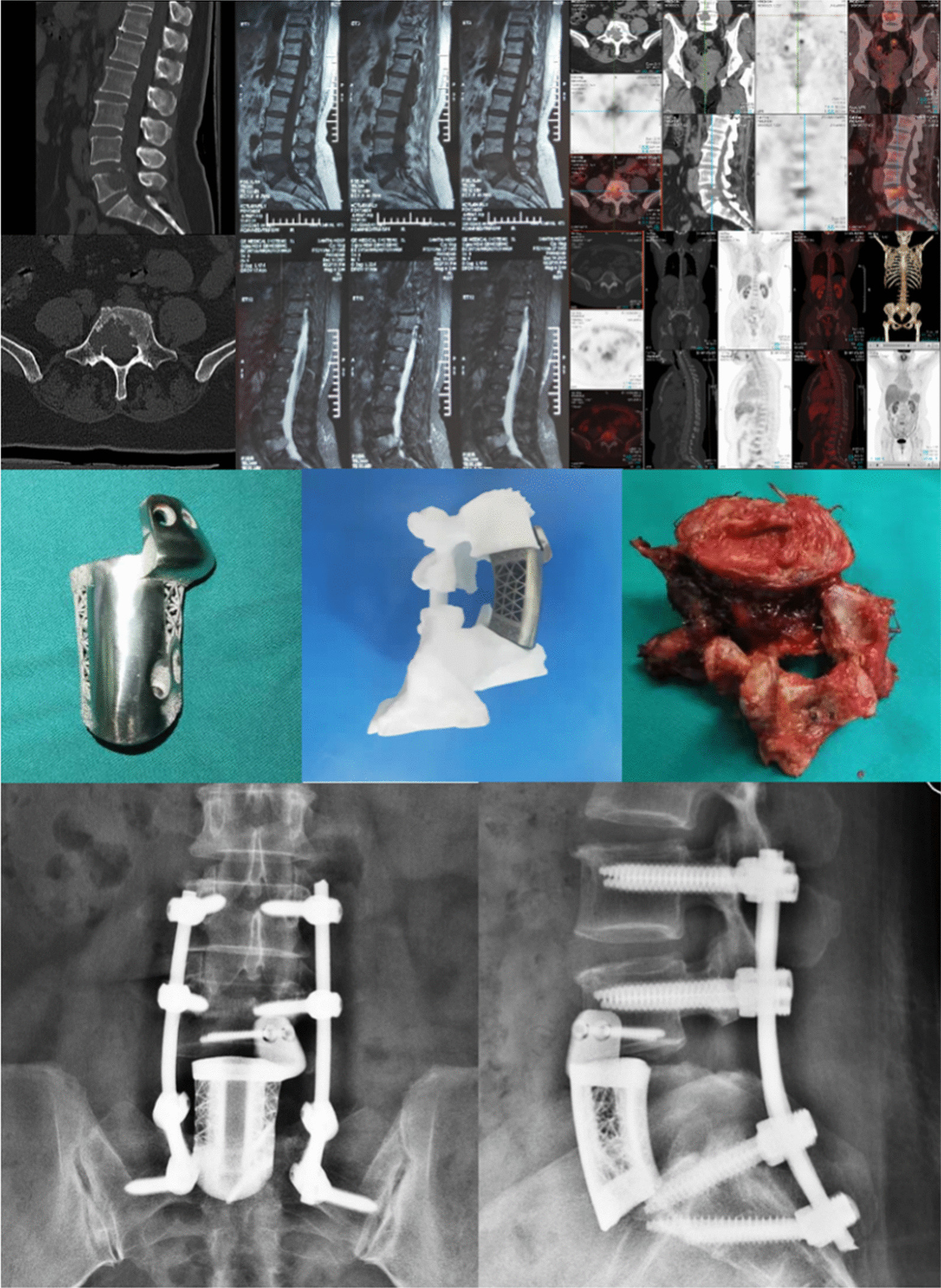
Case 2

*Case* 2: A 52-year-old female with a history of right breast cancer presented with 2 weeks of low back pain and numbness in the right lower extremity. Examination revealed nerve compression, and further diagnostic imaging, including CT, MRI, and PET-CT, confirmed spinal metastasis of breast cancer. The patient underwent en bloc resection of the tumor vertebral body with neuroelectrophysiological detection, followed by reconstruction using pedicle screw internal fixation and 3D-printed prosthesis implantation. The patient showed a favorable recovery after the surgery (Fig. 3).

## Discussion

The spine has now become the third most common site of tumor metastasis after the liver and lungs. Surgical resection remains the primary treatment for spinal tumors, involving tumor removal, spinal cord decompression, and stability reconstruction which are fundamental aspects of whole spine resection. Since the entire vertebral segment, along with ligament and muscle tissues, is removed during the procedure, the anterior, middle, and posterior pillars are disrupted, necessitating stability reconstruction [8]. The objective of reconstruction is to achieve long-term biological fusion. While titanium cages have provided effective support, concerns have arisen about their long-term reliability. Chen [9] followed up with 300 cases of cervical titanium cage reconstruction and observed that titanium cage subsidence was a common phenomenon associated with postoperative complications. Yoshioka [10] also found that internal fixation failure after total spinal resection of the thoraco-lumbar spine was a common occurrence. Due to the subsidence of the titanium cage and the collapse of the intervertebral space, maintaining the segment angle becomes challenging, leading to loss of biological stability, concentration of rod stress, and an increased risk of fracture. Park [11] identified perioperative radiotherapy as an independent risk factor for postoperative rod fracture, as radiotherapy affects bone mass and bone fusion, having a negative impact on spine stability. Moreover, the small contact surface of the titanium cage may cause the spinal force line to deviate from the bone contact surface, resulting in an unfavorable angle for bone fusion and internal fixation failure. However, artificial vertebral bone offers a larger contact surface, conducting the force line through the vertebral body via facial contact, which provides better stability [12].

Biological fusion is essential for maintaining long-term stability in spine reconstruction. The porous structure inside the 3D-printed artificial vertebra facilitates tissue fluid flow, promotes bone cell migration and proliferation, and a porosity of 70–80% is considered ideal for bone penetration. The complex microscopic rough structure on the surface encourages the recruitment of anti-inflammatory factors and osteoblast differentiation, creating a special cellular environment for bone formation [13, 14]. McGilvray [15] conducted in vitro fusion experiments in sheep lumbar spines and found that porous titanium alloy vertebrae exhibited superior bone penetration compared to PEEK material. Similarly, the use of a titanium alloy cage in anterior cervical surgery has demonstrated better osseointegration rates [16]. CT scans during patient follow-up in the observation group showed bone ingrowth in the bone-prosthesis interface and excellent osseointegration ability.

The length of the 3D-printed vertebral body is determined based on preoperative CT data, ensuring an accurate match with bone defects and allowing for the setup of autofixation or lateral fixation devices to connect adjacent vertebrae, resulting in better stability. In comparison to modular artificial vertebrae with autostabilization devices, the 3D-printed artificial vertebral autostabilization device is more personalized, facilitating intraoperative procedures for different patients [17]. Its bone contact surface has a rough structure imitating trabecular pores, rather than a spike-like device that can embed in the bone surface and reduce subsidence of the artificial vertebral body. Mobbs [18] reconstructed C2 with individualized artificial vertebrae, and at the 9-month postoperative follow-up, there was no loosening or displacement of the prosthesis. Doyoung [19] achieved accurate hemisacral reconstruction using 3D-printed prosthesis, and one year after the operation, the prosthesis remained in good position, with osseointegration occurring with the surrounding bone.

This study demonstrates that en bloc resection and reconstruction with 3D-printed artificial vertebrae offer advantages such as shorter operation time, reduced bleeding, and faster patient recovery. Compared to traditional titanium cages, 3D-printed artificial vertebrae maintain segment height more effectively and have a lower settlement rate. Their porous structure and rough bone contact surface are more conducive to osteogenesis. However, this study is limited by the number of cases and lacks long-term follow-up. Future research is needed to improve the therapeutic efficacy of spinal metastases.
